# Observation of acoustic spin

**DOI:** 10.1093/nsr/nwz059

**Published:** 2019-05-11

**Authors:** Chengzhi Shi, Rongkuo Zhao, Yang Long, Sui Yang, Yuan Wang, Hong Chen, Jie Ren, Xiang Zhang

**Affiliations:** 1 NSF Nano-scale Science and Engineering Center (NSEC), University of California, Berkeley, Berkeley, CA 94720, USA; 2 George W. Woodruff School of Mechanical Engineering, Georgia Institute of Technology, Atlanta, GA 30332, USA; 3 Center for Phononics and Thermal Energy Science, China-EU Joint Center for Nanophononics, Shanghai Key Laboratory of Special Artificial Microstructure Materials and Technology, School of Physics Sciences and Engineering, Tongji University, Shanghai 200092, China; 4 Faculties of Science and Engineering, University of Hong Kong, Hong Kong, China

**Keywords:** acoustic spin, spin-induced torque, spin–momentum locking

## Abstract

Unlike optical waves, acoustic waves in fluids are described by scalar pressure fields, and therefore are considered spinless. Here, we demonstrate experimentally the existence of spin in acoustics. In the interference of two acoustic waves propagating perpendicularly to each other, we observed the spin angular momentum in free space as a result of the rotation of local particle velocity. We successfully measured the acoustic spin, and spin-induced torque acting on a designed lossy acoustic probe that results from absorption of the spin angular momentum. The acoustic spin is also observed in the evanescent field of a guided mode traveling along a metamaterial waveguide. We found spin–momentum locking in acoustic waves whose propagation direction is determined by the sign of spin. The observed acoustic spin could open a new door in acoustics and its applications for the control of wave propagation and particle rotation.

## INTRODUCTION

The spin angular momentum describes the rotation of a vector field [1,2]. It provides an extra degree of freedom for the control of wave propagation and wave–matter interactions. The spin angular momentum of light is a result of the rotation of electric polarization [3]. In addition to the longitudinal spin represented by circularly polarized light where the axis of rotation is parallel to the propagation direction [4], the recently studied optical transverse spin [5–7] with the axis of rotation perpendicular to the direction of propagation has shown interesting physics such as strong spin–orbital interaction and the quantum spin Hall effect [8–17].

In fluids such as air and water, because the acoustic wave can be deterministically described by the scalar pressure field [18], the spin degree of freedom in acoustics has not been explored [19–22]. Recent studies have raised the question of whether acoustic spin can ever exist [19–22]. Similar to optical spin, one may consider acoustic spin as the rotation of the wave polarization given by its local particle velocity, but an acoustic plane wave propagating in free space is a longitudinal wave whose particle velocity always oscillates along the propagation direction and does not rotate [18]. Note that the spin angular momentum is different from the orbital angular momentum observed in acoustic vortices representing the circulation of energy flux [19–22] or helically shaped acoustic or optical beams associated with the twisted wavefront [23–29]. The spin angular momentum results from the rotation of polarization characterized by particle velocity field vector, while the orbital angular momentum comes from the spatial circular pattern of the wave field with non-zero curl.

**Figure 1.**
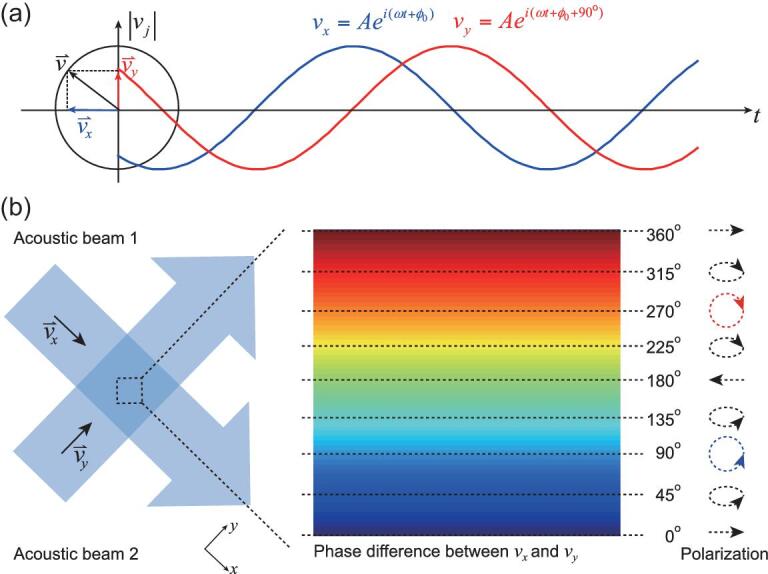
Acoustic spin as a rotating particle velocity field. (a) A rotating particle velocity (black arrow) can be decomposed into two components ${v_x}$ (blue arrow) and ${v_y}$ (red arrow) along the *x* and *y* directions. The two components shown as the blue and red lines are 90° out-of-phase. (b) Acoustic spin in the interference of two acoustic beams. Two beams with equal amplitudes propagating along the *x* and *y* directions contribute ${v_x}$ and ${v_y}$ components of the particle velocity field, respectively. The phase difference between ${v_x}$ and ${v_y}$ is a function of the position. The enlarged region shows an area where the phase difference between ${v_x}$ and ${v_y}$ changes from 0–360°. When the phase difference is 90° or 270° (equivalent to −90°), the local particle velocity field is rotating circularly, resulting in a spin-up (blue) or spin-down (red) acoustic field; when the phase difference is 0, 180° or 360°, the particle velocity field is oscillating along a line. In other cases, the local particle velocity rotates elliptically.

Here, we report the existence of spin angular momentum in airborne acoustics characterized by the rotation of local particle velocity, which cannot be characterized by the scalar pressure field of acoustic waves but must be analyzed using the local particle velocity field. A spinning local particle velocity ${\raise5pt\hbox{${\scriptstyle\rightharpoonup}$}{v}}$ can be decomposed into two perpendicular components ${v_x}$ and ${v_y}$ that are the same in amplitude but with a 90° difference in phase. The local particle velocity rotates clockwise or counterclockwise circularly depending on the relative phase difference (Fig. 1a). For convenience, we define the clockwise or counterclockwise acoustic spin as spin up or spin down, respectively. This rotating particle velocity field can be observed in the interference of two beams with equal amplitudes propagating perpendicularly to each other (Fig. 1b). Each beam contributes a component to the particle velocity field (${v_x}$ or ${v_y}$) in the interference pattern. The phase difference between these two orthogonal components is determined by the position, resulting in a spin-up or spin-down region respectively.

## GENERATION OF ACOUSTIC SPIN

To quantify the strength of the acoustic spin, we define the angular momentum carried by the spinning acoustic field in a unit volume as ${\raise5pt\hbox{${\scriptstyle\rightharpoonup}$}{s}} = {\rm{\ Im}}( {{\rho _0}{\raise5pt\hbox{${\scriptstyle\rightharpoonup}$}{v}}^{\,\,*} \times {\raise5pt\hbox{${\scriptstyle\rightharpoonup}$}{v}}})/2\omega $, where ${\rho _0}$ is the density of air and $\omega $ is the frequency of the acoustic field, which is derived from the acoustic angular momentum separating from the orbital angular momentum (see online Supplementary Material). We refer to ${\raise5pt\hbox{${\scriptstyle\rightharpoonup}$}{s}}$ as spin density. Similar angular momentum density was discussed for the total angular momentum carried by sound pulses [30,31]. The non-zero cross-product of the complex conjugate particle velocity with itself characterizes the rotation of the particle velocity field. For a circularly rotating particle velocity field, the spin density reaches its maximum value and represents the strongest angular momentum. For a linearly oscillating velocity field, both the spin density and the angular momentum are zero.

**Figure 2.**
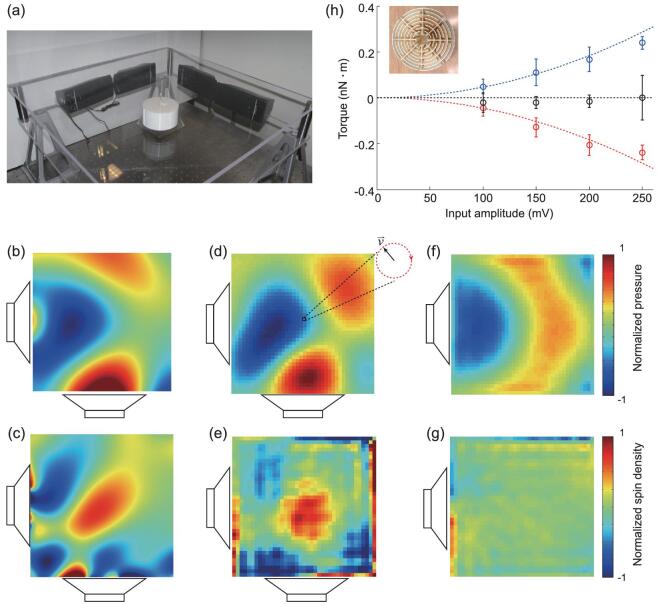
Experimental observation of acoustic spin. (a) Experimental set-up for the measurement of acoustic spin resulting from the interference of two perpendicular beams and the spin-induced torque acting on a coiled-space acoustic meta-atom. Two pairs of high-power speakers at two neighboring edges emit at 870 Hz with a 90° phase difference. The transparent glass walls at the top and bottom confine the acoustic waves propagating at the fundamental mode, mimicking an ideal 2D scenario extended infinitely in the perpendicular direction. The coiled-space meta-atom is hung at the center using a thin copper wire. (b) Simulated and (d) measured pressure fields show a 90° phase difference at the center of the interference pattern where the local particle velocity rotates clockwise, resulting in a spin-up field. (c) Simulated and (e) measured spin density distributions show that the spin density reaches its local maximum at the center where the meta-atom is located. (f) Measured pressure field when only one speaker is on. (g) Measured spin density when only one speaker is on. No spin exists in this case. The measured area for (d, e, f, g) is 40 × 40 cm, which is an enlargement of the theoretically calculated field in (c). The large spin density values at the exterior boundaries in (e) and (g) result from the sudden change of the air cross section area at the waveguide boundaries. The theoretically calculated spin density distribution in (c) does not exhibit large spin density at the exterior boundaries. (h) Measured torques acting on the coiled-space meta-atom (inset) versus input voltage amplitude loaded on the speakers. The spin-up or spin-down acoustic wave applies a negative or positive torque (with respect to the *z* axis in Fig. 1b) to the particle. The torques induced by the spin-up and spin-down waves are equal in amplitude and follow a quadratic relation with the input amplitude, in agreement with our theoretical prediction that the torque is proportional to the spin density.

In experiment, two acoustic beams are excited by two speakers placed at two neighboring sides of the set-up (Fig. 2a). By measuring the time-dependent pressure field *p* (Fig. 2d), the local particle velocity is given by ${\raise5pt\hbox{${\scriptstyle\rightharpoonup}$}{v}} = {\rm{\ }} - \nabla p/i\omega {\rho _0}$, where *i* is the imaginary unit. We found that the velocity rotates clockwise at the center, resulting in a spin-up acoustic field (Fig. 2d and Supplementary Material Movies 1 and 2). The measured pressure field and spin density shown in Fig. 2d and e agree with our simulations (Fig. 2b and c). In contrast, in the acoustic field excited only by one speaker, no spin is observed (Fig. 2f and g).

## SPIN-INDUCED TORQUE

The acoustic spin carries angular momentum, which can induce a torque through spin–matter interaction. In our study, an acoustic meta-atom that can support a dipole resonance—with the air coming out from half of the meta-atom and flowing into another half at a certain moment—based on a coiled-space structure [32] is placed in a spinning particle velocity field. Because the meta-atom is lossy due to the mechanical deformation of the meta-atom and the viscosity of air when the gas goes in and out through the tiny slits, the excitation of the dipole moment is always slightly delayed in phase compared to the exciting velocity field [30], which means that the excited dipole moment is not parallel with the exciting velocity field. Because an acoustic dipole tends to align with the velocity field, the misalignment drives the meta-atom, which provides torque acting on it. In other words, the meta-atom obtains angular momentum from the acoustic waves by absorbing the spin angular momentum.

To measure the spin-induced torque, we made a coiled-space meta-atom (inset of Fig. 2h and Supplementary Material Section II), which supports a dipole resonance at 870 Hz. The meta-atom is designed to be symmetric with a cylindrical shape to eliminate any possible torque due to the geometry itself. It is designed to be subwavelength in diameter so as to represent a probe to interact with the local spin. The meta-atom is hung by a thin copper wire at the center of the interference fields (Fig. 2a). A mirror is attached to the meta-atom to reflect a laser beam onto a ruler, which converts the rotation of the meta-atom into the deviation of the laser spot. The value of the torque is obtained by multiplying the torsional spring constant with the measured rotation angle (Supplementary Material Section III).

The torques induced by spin-up and spin-down acoustic waves are of equal amplitude along the –*z* and +*z* directions, respectively (Fig. 2h). The measured torques follow a quadratic relation with the amplitude of the input voltage loaded on the speakers, which shows that the spin-induced torque is linearly proportional to the spin density as predicted (Supplementary Material Section V). The noise is mainly contributed by environmental random vibrations. As a control experiment, the torque from one acoustic beam alone is undetectable, which means that a single beam without interference does not carry angular momentum. Note that the torque in this work originating from the delayed dipole resonance is fundamentally different from the acoustic viscous torque resulting from rotating acoustic particle velocity fields in viscous fluids [33–37]. The estimated viscous torque based on the theory described in [34] is one order smaller than the measured torque shown in Fig. 2h. The torque observed here can exist in non-viscous fluids as long as the meta-atom itself is lossy and can be polarized by the particle velocity field.

## SPIN–MOMENTUM LOCKING

**Figure 3.**
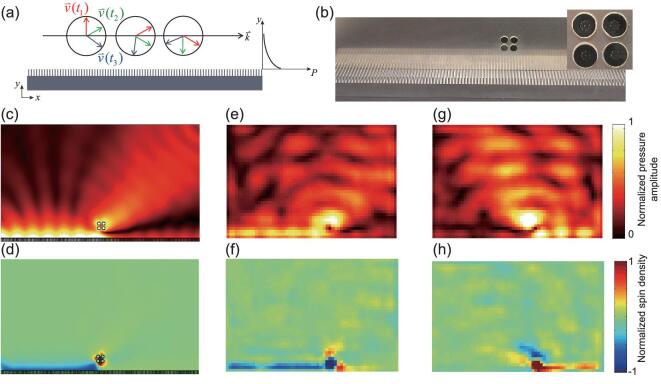
Spin–momentum locking in acoustics. (a) Schematic of the local particle velocity field of an evanescent acoustic wave supported by a metamaterial waveguide composed of periodic grooves. The acoustic field outside the waveguide decays exponentially along the *y* axis and propagates along the *x* axis. ${\raise5pt\hbox{${\scriptstyle\rightharpoonup}$}{v}}\,\,({{t_1}})$ (red), ${\raise5pt\hbox{${\scriptstyle\rightharpoonup}$}{v}}\,\,({{t_2}})$ (green) and ${\raise5pt\hbox{${\scriptstyle\rightharpoonup}$}{v}}\,\,({{t_3}})$ (blue) represent the particle velocities at times ${t_1}$, ${t_2}$ and ${t_3}$ with $0 < {t_1} < {t_2} < {t_3}$, respectively. The local particle velocity rotates clockwise in time. The propagation direction is solely determined by the spin direction, resulting in spin–momentum locking. (b) Experimental set-up for demonstrating the spin–momentum locking. Four mini-speakers (inset) are mounted near the acoustic waveguide. A rigid wall on each side confines the acoustic wave propagating in-plane. The four speakers emit at 2 kHz with a 90° phase difference between the neighboring speakers, mimicking a rotating acoustic dipole, which excites a spin-up or spin-down acoustic wave determined by the relative phase difference among the four speakers. (c, e, g) Normalized amplitudes of simulated and measured pressure fields. The acoustic metamaterial waveguide is at the bottom of each figure (not shown). The spin-down and spin-up acoustic waves are excited in (c, e) and (g), respectively. The measured area of (e) and (g) is 60 × 40 cm. The spin-down acoustic wave propagates only towards the left, while the spin-up wave propagates towards the right, demonstrating the phenomenon of spin–momentum locking. A thermal color scale is used for the pressure amplitude. (d, f, h) Normalized spin density for spin-down and spin-up acoustic waves in (c), (e) and (g), respectively, confirming the acoustic spin–momentum locking. A jet color scale is used for the spin density.

Spin–momentum locking is one of the most interesting physics used for chiral quantum circulators and asymmetric wave transport [8–17] in optics. We have also observed the acoustic spin–momentum locking phenomenon experimentally. In the acoustic waves supported by a metamaterial waveguide composed of periodic grooves, the evanescent field propagates along the waveguide but decays exponentially in the perpendicular direction (Fig. 3a). The two components of the particle velocity satisfy ${v_x} = {\rm{\ }} \pm ik{v_y}/\tau $, where *k* is the wave number along the waveguide and $\tau $ is the decaying constant in the perpendicular direction (Supplementary Material Section VI). Therefore, the *x* and *y* components of the particle velocity are innately 90° out-of-phase everywhere. The sign determines that the propagation direction is locked with the spin direction. The spin-up wave propagates to the right and the spin-down wave to the left.

The acoustic spin–momentum locking is demonstrated experimentally with the metamaterial waveguide confined by two rigid walls (Fig. 3b). Four mini-speakers are packed together and mounted in the vicinity of the metamaterial waveguide with their phases modulated 90° relative to each other to mimic a rotating acoustic dipole source (Supplementary Material Section VII). A similar experimental set-up can be used to observe the ‘spin Hall-like effect’ of orbital angular momentum carrying acoustic waves in hyperbolic metamaterials [38]. When this dipole source rotates counterclockwise, it excites the spin-down acoustic wave that propagates only towards the –*x* direction (Fig. 3c (simulation) and e (experiment)). On the other hand, the clockwise rotating dipole source excites the spin-up acoustic wave propagating only towards the +*x* direction (Fig. 3g). The particle velocity fields in Supplementary Material Movies 3 and 4 also show that the acoustic wave with counterclockwise/clockwise rotating particle velocity propagates towards the –*x*/+*x* direction. The calculated and measured spin densities further confirm the spin–momentum locking phenomenon (Fig. 3d, f and h).

## CONCLUSIONS

In conclusion, we have demonstrated the existence of spin in acoustics. The spin-induced torque is measured to be proportional to the spin density and the spin–momentum locking is shown. The observation of acoustic transverse spin provides a fundamental platform for future studies on acoustic spin physics such as spin–orbital interaction, acoustic spin Hall effect, and spin-induced non-reciprocal acoustic physics that are important for applications in the control of wave propagation and particle rotation.

## Supplementary Material

nwz059_Supplemental_Files

## References

[1] Belinfante FJ . On the spin angular momentum of mesons. Physica 1939; 6: 887–98.

[2] Ohanian HC . What is spin? Am J Phys 1986; 54: 500–5.

[3] Belinfante FJ . On the current and the density of the electric charge, the energy, the linear momentum and the angular momentum of arbitrary fields. Physica 1940; 7: 449–74.

[4] Andrews DL , Babiker M. The Angular Momentum of Light. Cambridge: Cambridge University Press, 2013.

[5] Bliokh KY , Bekshaev AY, Nori F. Extraordinary momentum and spin in evanescent waves. Nat Commun 2014; 5: 3300. 24598730 10.1038/ncomms4300

[6] Bekshaev AY , Bliokh KY, Nori F. Transverse spin and momentum in two-wave interference. Phys Rev X 2015; 5: 011039.

[7] Aiello A , Banzer P, Neugebauer M et al. From transverse angular momentum to photonic wheels. Nat Photon 2015; 9: 789–95.

[8] Rodriguez-Fortuno FJ , Marino G, Ginzburg P et al. Near-field interference for the unidirectional excitation of electromagnetic guided mode. Science 2013; 340: 328–30. 23599487 10.1126/science.1233739

[9] Petersen J , Volz J, Rauschenbeutel A. Chiral nanophotonic waveguide interface based on spin-orbit interaction of light. Science 2014; 346: 67–71. 25190718 10.1126/science.1257671

[10] Bliokh KY , Smirnova D, Nori F. Quantum spin Hall effect of light. Science 2015; 348: 1448–51. 26113717 10.1126/science.aaa9519

[11] Bliokh KY , Nori F. Transverse and longitudinal angular momenta of light. Phys Rep 2015; 592: 1–38.

[12] Bliokh KY , Rogriguz-Fortuno FJ, Nori F et al. Spin-orbit interactions of light. Nat Photon 2015; 9: 796–808.

[13] Lodahl P , Mahmoodian S, Stobbe S et al. Chiral quantum optics. Nature 2017; 541: 473–80. 28128249 10.1038/nature21037

[14] Shomroni I , Rosenblum S, Lovsky Y et al. All-optical routing of single photons by a one-atom switch controlled by a single photon. Science 2014; 345: 903–6. 25146283 10.1126/science.1254699

[15] Sollner I , Mahmoodian S, Hansen SL et al. Deterministic photon-emitter coupling in chiral photonic circuits. Nat Nanotechnol 2015; 10: 775–8. 26214251 10.1038/nnano.2015.159

[16] Rosenblum S , Bechler O, Shomroni I et al. Extraction of a single photon from an optical pulse. Nat Photon 2016; 10: 19–22.

[17] Scheucher M , Hilico A, Will E et al. Quantum optical circulator controlled by a single chirally coupled atom. Science 2016; 354: 1577–80. 27940579 10.1126/science.aaj2118

[18] Crocker MJ . Handbook of Acoustics. New York: Wiley, 1998.

[19] He C , Ni X, Ge H et al. Acoustic topological insulator and robust one-way sound transport. Nat Phys 2016; 12: 1124–9.

[20] Lu J , Qiu C, Ke M et al. Valley vortex states in sonic crystals. Phys Rev Lett 2016; 116: 093901. 26991176 10.1103/PhysRevLett.116.093901

[21] Zhang Z , Wi Q, Cheng Y et al. Topological creation of acoustic pseudospin multipoles in flow-free symmetry-broken metamaterial lattice. Phys Rev Lett 2017; 118: 084303. 28282192 10.1103/PhysRevLett.118.084303

[22] Marston PL . Humblet's angular momentum decomposition applied to radiation torque on metallic spheres using the Hagen-Rubens approximation. J Quant Spectrosc Radiat Transfer 2018; 220: 97–105.

[23] Hefner BT , Marston PL. An acoustical helicoidal wave transducer with applications for the alignment of ultrasonic and underwater systems. J Acoust Soc Am 1999; 106: 3313–6.

[24] Brunet T , Thomas J-L, Marchiano R et al. Experimental observation of azimuthal shock waves on nonlinear acoustical vortices. New J Phys 2009; 11: 013002.

[25] Jiang X , Li Y, Liang B et al. Convert acoustic resonances to orbital angular momentum. Phys Rev Lett 2016; 117: 034301. 27472113 10.1103/PhysRevLett.117.034301

[26] Jiang X , Zhao J, Liu S-L et al. Broadband and stable acoustic vortex emitter with multi-arm coiling slits. Appl Phys Lett 2016; 108: 203501.

[27] Naify CJ , Rodhe CA, Martin TP et al. Generation of topologically diverse acoustic vortex beams using a compact metamaterial aperture. Appl Phys Lett 2016; 108: 223503.

[28] Ye L , Qiu C, Lu J et al. Making sound vortices by metasurfaces. AIP Adv 2016; 6: 085007.

[29] Miao P , Zhang Z, Sun J et al. Orbital angular momentum microlaser. Science 2016; 353: 464–7. 27471299 10.1126/science.aaf8533

[30] Lekner J . Angular momentum of sound pulses. J Phys: Condens Matter 2006; 18: 6149–58. 21690827 10.1088/0953-8984/18/26/032

[31] Lekner J . Energy and momentum of sound pulses. Physica A 2006; 363: 217–25.

[32] Cheng Y , Zhou C, Yuan BG et al. Ultra-sparse metasurface for high reflection of low-frequency sound based on artificial Mie resonances. Nat Mater 2015; 14: 1013–9. 26322718 10.1038/nmat4393

[33] Zhao R , Manjavacas A, de Abajo FJG et al. Rotational quantum friction. Phys Rev Lett 2012; 109: 123604. 23005949 10.1103/PhysRevLett.109.123604

[34] Wang TG , Kanber H, Rudnick I. First-order torques and solid-body spinning velocities in intense sound fields. Phys Rev Lett 1977; 38: 128–30.

[35] Busse FH , Wang TG. Torque generated by orthogonal acoustic waves— Theory. J Acoust Soc Am 1981; 69: 1634–8.

[36] Zhang L , Marston PL. Acoustic radiation torque on small objects in viscous fluids and connection with viscous dissipation. J Acoust Soc Am 2014; 136: 2917–21. 25480039 10.1121/1.4900441

[37] Bernard I , Doinikov AA, Marmottant P et al. Controlled rotation and translation of spherical particles or living cells by surface acoustic waves. Lab Chip 2017; 17: 2470–80. 28617509 10.1039/c7lc00084g

[38] Ju F , Cheng Y, Liu X. Acoustic spin Hall-like effect in hyperbolic metamaterials controlled by the helical wave. Sci Rep 2018; 8: 11113. 30042498 10.1038/s41598-018-29359-w PMC6057999

