# Supplementary material for: Observation of acoustic spin
Source: Natl Sci Rev. 2019 May 11;6(4):707–12. doi: 10.1093/nsr/nwz059 (PMC8291453; doi:10.1093/nsr/nwz059)
Supplement: nwz059_Supplemental_Files [file nwz059_supplemental_files.zip › Observation of Acoustic Spin_SI_final.pdf]

Observation of acoustic spin

Chengzhi Shi[1, 2†], Rongkuo Zhao[1†], Yang Long[3†], Sui Yang[1], Yuan Wang[1], Hong Chen[3], Jie Ren[3•], Xiang Zhang[1,4*]

[1]NSF Nano-scale Science and Engineering Center (NSEC), University of California, Berkeley, 3112 Etcheverry Hall, Berkeley, CA 94720, USA

[2]George W. Woodruff School of Mechanical Engineering, Georgia Institute of Technology, 003 Love Manufacturing Building, Atlanta, GA 30332, USA

[3]Center for Phononics and Thermal Energy Science, China-EU Joint Center for Nanophononics, Shanghai Key Laboratory of Special Artificial Microstructure Materials and Technology, School of Physics Sciences and Engineering, Tongji University, Shanghai 200092, China

[4]Materials Science Division, Lawrence Berkeley National Laboratory, 1 Cyclotron Road, Berkeley, CA 94720, USA

[†]These authors contributed equally to this work.

[•]*Email:* *xonics@tongji.edu.cn*

[*]*Email:* *xiang@berkeley.edu*

I. Derivation of Acoustic Orbital and Spin Angular Momentum

In acoustics, the energy transport is characterized by the time averaged Poynting momentum vector $\vec{m}$, which is the momentum density of acoustic wave. For harmonic acoustic waves, the momentum density is given by

$$\vec{m} = \frac{1}{2c^2}\mathrm{Re}[p^*\vec{v}] = \frac{1}{2c^2}\mathrm{Re}\left[\frac{i\rho_0 c^2}{\omega}(\nabla\cdot\vec{v}^*)\vec{v}\right] = \frac{\rho_0}{2\omega}\mathrm{Im}[(\nabla\cdot\vec{v})\vec{v}^*], \qquad \text{(S1)}$$

where p is the pressure field, $\vec{v}$ is the local particle velocity field, c is the sound speed in air, ρ_0 is the density of air, and ω is the frequency of the acoustic wave. Applying the following vector identities

$$\mathrm{Im}[\nabla \times (\vec{v}^* \times \vec{v})] = \mathrm{Im}[\vec{v}^*(\nabla \cdot \vec{v})] - \mathrm{Im}[\vec{v}(\nabla \cdot \vec{v}^*)] + \mathrm{Im}[(\vec{v} \cdot \nabla)\vec{v}^*] - \mathrm{Im}[(\vec{v}^* \cdot \nabla)\vec{v}]$$

$$= 2\mathrm{Im}[\vec{v}^*(\nabla \cdot \vec{v})] + 2\mathrm{Im}[(\vec{v} \cdot \nabla)\vec{v}^*] \ , \qquad \text{(S2)}$$

the right-hand side of Eq. (S1) can be separated into two terms

$$\vec{m} = \frac{\rho_0}{2\omega}\mathrm{Im}[(\nabla \cdot \vec{v})\vec{v}^*] = \frac{\rho_0}{2\omega}\mathrm{Im}[(\vec{v}^* \cdot \nabla)\vec{v}] + \frac{\rho_0}{4\omega}\mathrm{Im}[\nabla \times (\vec{v}^* \times \vec{v})] = \vec{m}_o + \vec{m}_s, \qquad \text{(S3)}$$

where $\vec{m}_o = \frac{\rho_0}{2\omega}\mathrm{Im}[(\vec{v}^* \cdot \nabla)\vec{v}]$ and $\vec{m}_s = \frac{\rho_0}{4\omega}\mathrm{Im}[\nabla \times (\vec{v}^* \times \vec{v})]$. The orbital angular momentum density of the acoustic wave is given by $\vec{l} = \vec{r} \times \vec{m}_o$, which can be used to calculate the total orbital angular momentum $\vec{L} = \int \vec{l} d\vec{r}^3 = \int (\vec{r} \times \vec{m}_o) d\vec{r}^3$. The total spin angular momentum of the acoustic wave is given by

$$\vec{S} = \int (\vec{r} \times \vec{m}_s) d\vec{r}^3 = \int \left(\vec{r} \times \frac{\rho_0}{4\omega}\mathrm{Im}[\nabla \times (\vec{v}^* \times \vec{v})]\right) d\vec{r}^3$$

$$= \frac{\rho_0}{2\omega}\mathrm{Im}[\int (\vec{r} \times \left[\frac{1}{2}\nabla \times (\vec{v}^* \times \vec{v})\right])] d\vec{r}^3 = \frac{\rho_0}{2\omega}\mathrm{Im}[\int (\vec{v}^* \times \vec{v}) d\vec{r}^3]. \qquad \text{(S4)}$$

The spin angular momentum density can be defined as $\vec{s} = \frac{\rho_0}{2\omega}\mathrm{Im}[\vec{v}^* \times \vec{v}]$. Eq. S4 shows that acoustic spin carries angular momentum in the absence of a momentum arm. Similar separation of angular momentum into spin and orbital parts can be found in optics [1-6]. This spin density can also be written as $\vec{s} = \rho_0 \langle \vec{v} | \vec{\sigma} | \vec{v} \rangle / 2\omega$, where $\vec{\sigma} = \begin{pmatrix} 0 & -i \\ i & 0 \end{pmatrix}$ is the spin operator in 2D space. The physical differentiation between the spin and orbital angular momentum is that a probe meta-atom rotates around the particle center when placed at an arbitrary location in the acoustic spin field, while the rotation of the same meta-atom is around the center of the acoustic field when the wave possesses orbital angular momentum.

II. Dipole Resonance of the Cylindrical Coiled Space Meta-Atom

Cylindrical coiled space meta-atom has the ability to slow sound speed and reduce the resonant frequency of dipole resonance [7]. However, previously design coiled space meta-atom is asymmetric along the azimuthal direction that can result in an applied torque when interact with acoustic plane waves [7]. To rule out this asymmetric effect, we design a symmetric coiled space meta-atom (Figure S1a) with its dipole mode shown in Figure S1b. This dipole resonance interacts with the rotating particle velocity of acoustic transverse spin through thermo-viscous effect. The full wave simulation of the transmission through the meta-atom considering the thermo-viscous effect [8] indicates that the dipole resonance occurs at 890 Hz (blue curve is Figure S1c). The experimental measurement of the transmission shows that this dipole resonance occurs at 870 Hz (black circles in Figure S1c), which is chosen as the frequency for the measurement of spin induced torque in Figure 2 of the main text to enhance the interaction.

III. Measurement of the Spin Induced Torque

Because the spin induced torque is small, we apply Cavendish's method to amplify measurement of the rotation of the meta-atom θ with a laser beam [9]. The spin induced torque is given by

$$\tau = \kappa\theta, \tag{S5}$$

where the rotational stiffness of the copper wire is given by

$$\kappa = (2\pi f_0)^2 I. \quad \text{(S6)}$$

Here, the rotational resonant frequency of the suspended meta-atom with the copper wire is measured from the frequency response of the free rotational vibration of the meta-atom $f_0 = 0.02$ Hz and the moment of inertia of the meta-atom is given by

$$I = \frac{mr^2}{2}, \quad \text{(S7)}$$

where the mass and the radius of the meta-atom are m = 1.4 g and r = 5 cm. When measuring the rotation of the meta-atom, rotational vibrations above 0.005 Hz are filtered to avoid the strong influence of the rotational resonance on the torque measurements. The rotational vibrations of the suspended meta-atom due environmental thermal fluctuation and ground vibration contribute to the measurement error.

IV. Response of the High Power Speakers Used for Torque Measurement

To characterize the effect of the high power speakers on the spin induced torque measurement, we perform the measurement of the response of the speakers with varying input voltage amplitude loaded on the speakers using a microphone (Figure S2). It can be observed that the speakers perform linearly before the saturation at 250 mV input voltage. Therefore, we choose the input voltage amplitude to be smaller than 250 mV for our spin induced torque measurement in Figure 2 of the main text.

V. Relations between the Torque, Input Amplitude, and Spin Density

As shown in Figure 2b of the main text, the measured torque and the input voltage

amplitude follow a quadratic relation. From Figure S2, we observe the input amplitude is linearly related to the microphone measured amplitude, i.e. the amplitude of the pressure field. According to Eq. (S12) in later discussion, the amplitude of the particle velocity is proportional to the pressure field. Thus, the measured torque and the amplitude of the particle velocity field follow a quadratic relation. Because the spin density is given by $\vec{s} = \mathrm{Im}(\rho_0 \vec{v}^* \times \vec{v})/2\omega$, the spin density and the amplitude of the particle velocity field also satisfy a quadratic relation. Therefore, the measured torque is proportional to the spin density.

It can be further confirmed that the torque we measured is dominated by spin matter interaction because the orbital angular momentum of the interference pattern is tiny compared with the spin angular momentum. As derived by Eqs. (S3) and (S4), the orbital angular momentum of the two interfering beams in Figure 2e over the area covered by the meta-atom for later torque measurement of the main text is

$$\vec{L} = \frac{\rho_0}{2\omega}\mathrm{Im}[\int (\vec{r} \times (\vec{v}^* \cdot \nabla)\vec{v}) d\vec{r}^3] = 6.3 \times 10^{-10}\ \ \mathrm{kg} \cdot \mathrm{m}^2/\mathrm{s}. \qquad \text{(S8)}$$

The spin angular momentum of the two interfering beams over the same area is given by

$$\vec{S} = \frac{\rho_0}{2\omega}\mathrm{Im}[\int (\vec{v}^* \times \vec{v}) d\vec{r}^3] = 8.4 \times 10^{-8}\ \ \mathrm{kg} \cdot \mathrm{m}^2/\mathrm{s}. \qquad \text{(S9)}$$

The origin is set at the center of the wave field where the meta-atom is placed to eliminate torque induced by offset of the acoustic beams. Thus, the orbital angular momentum of the measured interference pattern is within numerical error. The measure torque in Figure 2 of the main text is dominantly contributed by spin matter

interaction.

VI. Wave Supported by the Acoustic Metamaterial Waveguide

The acoustic wave supported by the periodic grooves serving as an acoustic metamaterial waveguide in Figure 3a of the main text is propagating along *x*-direction, while decaying evanescently along *y*-direction (Figure S3a). Therefore, the pressure field of the acoustic wave can be expressed as

$$p = Ae^{-\tau y}e^{i(\omega t-kx)}, \tag{S10}$$

where *i* is the imaginary unit, *A* is the amplitude of the pressure wave, ω is the angular frequency, *k* is the wave number along the propagation direction, and τ is the decaying constant perpendicular to the propagation direction. The particle velocity field is given by

$$\vec{v} = -\frac{1}{i\omega\rho_0}\nabla p, \tag{S11}$$

where ρ_0 is the air density. Thus, the two components of the particle velocity field are

$$v_x = -\frac{1}{i\omega\rho_0}(-ik)p, \qquad v_y = -\frac{1}{i\omega\rho_0}(-\tau)p. \tag{S12}$$

Thus, the two components of the particle velocity satisfy $v_x = ikv_y/\tau$, indicating they are 90 degrees out-of-phase and the particle velocity field is rotating.

This acoustic metamaterial waveguide has infinite effective density along the *x*-direction [10]. According to the derivation in [10], the dispersion relation of the acoustic wave propagating along the metamaterial waveguide satisfies

$$k = k_0\sqrt{1+\frac{w^2}{p^2}\tan^2(k_0T)}, \qquad k_0 = \omega\sqrt{\rho_y/\kappa_0}, \qquad \rho_y = (\frac{p}{w})\rho_0, \tag{S13}$$

where κ_0 is the bulk modulus of air, p = 6.35 mm is the period, w = 4.76 mm is the width, and T = 15.9 mm is the depth of the grooves (Figure S3b). This dispersion relation is similar to spoof surface plasma and 2 kHz which is below the cut-off frequency is chosen for the demonstration (Figure S3c).

VII. Rotating Acoustic Dipole Created by Four Speakers

The rotating acoustic dipole can be realized by modulating the phase relation of the four speakers in Figure 3b of the main text. This is done by tuning the phase of the speakers such that the neighboring speaker has a phase increased by 90 degrees in the clockwise direction as shown in Figure S4. In this case, the particle velocity field (red arrows in Figure S4) representing the direction of the acoustic dipole is also rotating in the clockwise direction. To realize a rotating dipole in the counterclockwise direction, we need to tune the speakers such that their phases decrease by 90 degrees in the counterclockwise direction.

References

[1] Berry, M. V., Optical currents, *J. Opt. A Pure Appl. Opt.* **11**, 094001 (2009).

[2] Belinfante, F. J., On the current and the density of the electric charge, the energy, the linear momentum and the angular momentum of arbitrary fields, *Physica* **7**, 449-474 (1940).

[3] Bliokh, K. Y., Bekshaev, A. Y., and Nori, F., Extraordinary momentum and spin in evanescent waves, *Nature Comm.*, **5**, 3300 (2014).

[4] Bekshaev, A. Y., Bliokh, K. Y., and Nori, F., Transverse spin and momentum in two-wave interference, *Phys. Rev. X* **5**, 011039 (2015).

[5] Bliokh, K. Y., and Nori, F., Transverse and longitudinal angular momentum of light, *Phys. Rep.*, **592**, 1-38 (2015).

[6] Bliokh, K. Y., Rogriguz-Fortuno, F. J., Nori, F., and Zayats, A. V., Spin-orbit interactions of light, *Nature Photonics*, **9**, 796-808 (2015).

[7] Cheng, Y., Zhou, C., Yuan, B. G., Wu, D. J., Wei, Q., and Liu, X. J., Ultra-sparse metasurface for high reflection of low frequency sound based on artificial Mie resonances, *Nature Mater.* **14**, 1013-1019 (2015).

[8] Ward, G. P., Lovelock, R. K., Murray, A. R. J., Hibbins, A. P., Sambles, J. R., and Smith, J. D., Boundary-layer effects on acoustic transmission through narrow slit cavities, *Phys. Rev. Lett.* **115**, 044302 (2015).

[9] Cavendish, H., Experiments to determine the density of the Earth, *Philosophical Trans. Royal Soc. London* **88**, 469-526 (1798).

[10] Zhu, J., Chen, Y., Zhu, X., Garcia-Vidal, F. J., Yin, X., Zhang, W., and Zhang, X., Acoustic rainbow trapping, *Sci. Rep.* **3**, 1728 (2013).

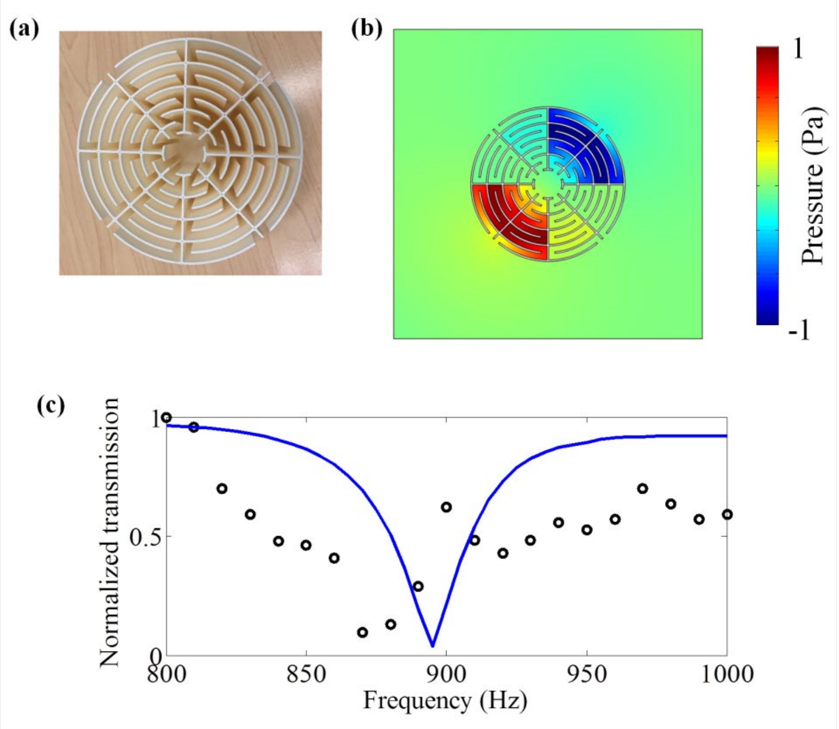

Figure S1: Symmetric coiled space meta-atom. (a) The meta-atom fabricated by 3D printer with ABS plastic. (b) The pressure field of the dipole mode shape of the meta-atom. (c) Calculated with thermo-viscous effect (blue curve) [7] and measured (black circles) of the normalized transmission through the meta-atom. From the calculation, the dipole resonance occurs at 890 Hz. The experimental result shows that this dipole resonance occurs at 870 Hz, which is chose for the measurement of the spin induced torque.

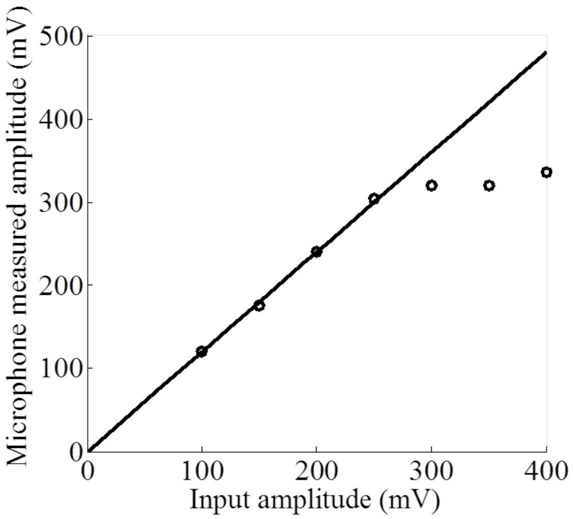

Figure S2: Speaker response with varying input voltage amplitude of the high power speakers used for spin induced torque measurement. A microphone is fixed at a location far away from the speakers to avoid the saturation of the microphone response. The speakers behave linearly before the saturation occurs at 250 mV input voltage.

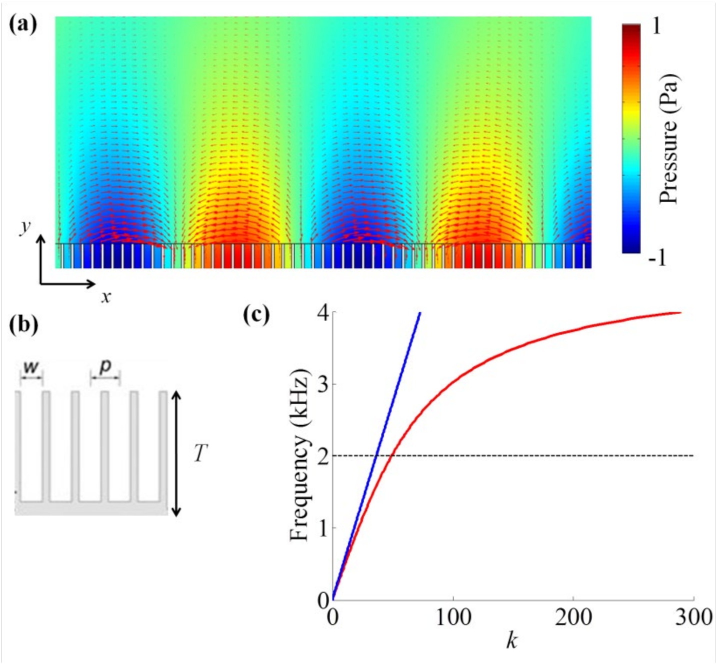

Figure S3: Acoustic wave propagating along the metamaterial waveguide. (a) The pressure field (jet color scale) and particle velocity field (red arrows) of the acoustic wave propagating along the metamaterial waveguide. The wave is propagating along *x*-direction, while evanescently decaying along *y*-direction. (b) Geometry of the grooves. The period is p = 6.35 mm, width w = 4.76 mm, and depth T = 15.9 mm. (c) The dispersion relations of the metamaterial waveguide (red) and sound cone (blue).

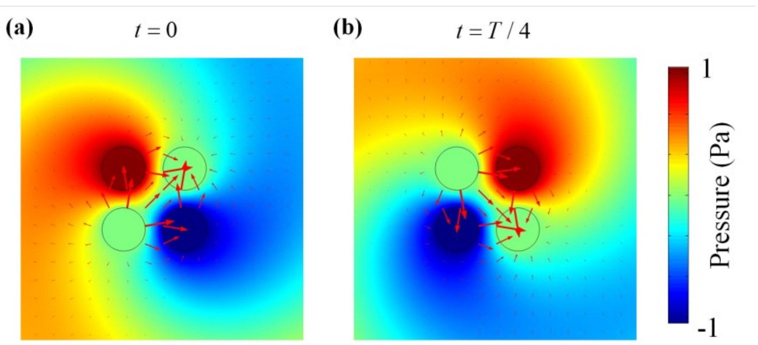

Figure S4: Acoustic rotating dipole mimicked by four speakers. (a, b) the pressure field (jet color scale) and particle velocity field (red arrows) of the four speakers emitting at 2 kHz with phase increasing by 90 degrees between the neighboring speakers in the clockwise direction at time 0 in (a) and a quarter period in (b), respectively. The acoustic dipole represented by the particle velocity direction is rotating in the clockwise direction.
